# Synthesis and characterization of plant extracted silver nanoparticles and advances in dental implant applications

**DOI:** 10.1016/j.heliyon.2022.e12313

**Published:** 2022-12-10

**Authors:** Nayem Hossain, Mohammad Aminul Islam, Mohammad Asaduzzaman Chowdhury

**Affiliations:** a Department of Mechanical Engineering IUBAT-International University of Business Agriculture and Technology, Bangladesh; b Department of Mechanical Engineering Dhaka University of Engineering and Technology (DUET), Gazipur Gazipur-1707, Bangladesh

**Keywords:** Synthesis, Plant extract, Ag-NP'S (silver nanoparticle), Characterization, Dental implant

## Abstract

Dental implantology has always emphasized silver nanoparticles (AgNPs) for various applications due to their biocompatibility, antibacterial activity, and increased surface volume ratio offered by these particles. It is utilized to a large extent in the dental implant industry as a surface modification, biocompatible constituent and composite material. AgNPs may be produced inexpensively, sustainably, and environmentally responsibly by utilizing technologies that extract the plant material. The phytochemical components that are contained in plants make them a better, non-toxic, and more cost-effective alternative to both physical and chemical approaches. Because the size and shape of AgNP depend on their synthesis method and technique, and because the efficacy and toxicity of AgNP depend on both size and shape, synthesis methods and techniques have recently become the focus of a significant amount of research attention. In this review, we discussed Plant Extracted Ag-NP's whose sizes range up to 100nm. This review also focuses on recent research advancements in the Plant Extracted synthesis of AgNPs, as well as their characterization methodologies, current obstacles, future possibilities, and applications in dental implantology.

## 1 Introduction

The utilization of nanoparticles lies at the heart of nanotechnology, which is sometimes defined as a science that spans several disciplines [1].The Physio-chemical characteristics of metals vary as they approach the nanoscale, and the properties of nanoparticles are distinct from those of bulk metal [2].Due to their unique physical and biochemical characteristics in contrast to their macro- and micro-complements, Ag-NP's have attracted interest among the numerous nanomaterials now in use [3].The availability of increased surface area to volume ratios is the primary factor contributing to the high efficiency of silver nanoparticles [4].

Physical, chemical, and biological methods can all be used to create Ag-NP's. The chemical approach is poisonous and expensive, and the physical methods take a lot of energy to sustain the high pressure and temperature needed for the reaction [5].Nanomaterials' drawbacks, such as their toxicity toward bone cells, their variable biocompatibility depending on size, surface, and composition, and their high price, have prompted the development of biochemical methods or biosynthesis, such as the use of biomolecular extracts from plants [6, 7].The availability of a wide variety of metabolites with great reduction potentials, worldwide distribution, safe handling, low waste and energy costs, and large and accessible reserves make plant extracts very popular [8, 9].

Size, crystalline structure, composition, and other physical properties of nanoparticles have all been determined through various methods of study.In many situations, assessing physical characteristics requires utilizing more than one method.Since each approach has its advantages and disadvantages, picking the right one can be challenging; a combinatorial characterization is often necessary.Characterizing NPs requires addressing important parameters such as size and form [10, 11, 12].The success of Nps in different contexts hinges on how well we can characterize them.Many different methods exist for characterizing substances.

In dentistry, silver nanoparticles are used to make antibacterial chemicals that improve dental implants. They can be used in conjunction with acrylic resins for fabricating removable dentures during prosthetic treatments, composite resins for direct restoration during restorative treatments, endodontic irrigants and obturation materials during endodontic procedures, orthodontic adhesives, and titanium coating during dental implant procedures [13].A quantitative study found that small Ag-NPs inhibit antibacterial activity by releasing more Ag-NP ions. Nanoparticles' antimicrobial properties can be improved and more work can be done in improving those certain characteristics [14]. Aggregation of Ag-NPs decreases their surface area to volume ratio and activity in numerous areas (antimicrobial, catalyst, etc.). The phytochemical method can fix this issue since it contains potent reducing and capping agents. The future of phytochemical nanoparticle synthesis rests on the genetic engineering of plant genomes to improve the production of reduction and capping agents [1]. Nanoparticle release from customized implants can elicit a cytotoxic response, although few efforts have been made to produce nano-engineered coverings with adequate mechanical stability [15, 16, 17]. Our main objective in this review is to discuss the use and effectiveness of Ag–Np in dental implants, the synthesis processes related to shape, size, and toxicity, its effects, an improvement on dental implants, and why plant extraction is preferable in this regard. We will also discuss potential future research into using plant-extracted Ag-Nps more effectively and engagingly in dental implantology.

## 2 Importance of Ag-NP's in dental implants

Peri-implantitis, brought on by the buildup of bacterial biofilm on dental implant surfaces, is a leading cause of implant failure. Altering the nanotopography of a surface is proposed to reduce bacterial attachment to implants [18, 19]. Because of AgNP's exceptional antibacterial qualities, they are one of the most popular alternatives for use in dental implant doping and repair [13]. Ag electrostatically binds to the cytoplasmic membrane and the bacterial cell wall, disrupting the cellular structure of the bacterium [15, 20]. Surface modification with Ag NPs is highly recommended for dental implants because of their ability to promote osteogenesis and soft-tissue integration [21]. To provide synergistic antibacterial (*S. epidermidis*, *S. mutans*, and *E. coli*) and osteogenic (human osteoblast-like cells, SAOS-2) properties, Ag-NP's have been deposited on Ti implants via anodic spark deposition [22]. Ag-NP's did not show any signs of cytotoxicity when deposited on sand-blasted, big grit, and acid-etched titanium, but they did limit the growth of*S.aureus* and *F. nucleatum*. These findings imply that titanium implants coated with Ag-NP's can be provided with complementary antibacterial and osteogenic capabilities, which bodes well for their secure and long-term therapeutic uses [23, 24]. Against *S. aureus* and *P. aeruginosa*, Ag NPs extracted from plants showed bactericidal activity [25].Because Ag-NP's have such distinctive properties, there is a significant amount of interest in utilizing them in dental implants.

## 3 Synthesis processes

Both bottom-up and top-down methods can be used for the synthesis of Ag-NP's [26]. Using a top-down methodology, Ag-NP's are produced using a variety of physical techniques that scale down the starting material from bulk to nanoscale [27]. Metal NPs' physicochemical characteristics are mostly determined by their surface structure, which varies greatly from case to case [28]. The high temperature and pressure settings necessary for the synthesis process need a great deal of energy, which is another disadvantage of this method [28, 29]. Several processes, including thermolysis, pyrolysis, radiation-induced, and lithography, fall under this category [28, 29, 30].

The self-assembly technique is a name for the bottom-up strategy [31]. These techniques rely largely on the assemblage of produced NPs into a final nanomaterial of the required size via different biological and chemical processes [32]. For a reasonable price, this approach offers a considerably increased possibility of fabricating Ag-NP's with improved chemical compositional homogeneity and reduced surface imperfection [32]. The necessity for nonpolar organic solvents, toxic chemicals, synthesized capping agents, and some other stabilizing agents severely restrict the biological applications of these chemical techniques [32, 33, 34].

Research teams are aimed at developing greener, safer, more effective, and more compatible alternatives to traditional chemical synthesis [35]. As a result, scientists have been focusing on the biosynthesis technique for creating Ag-NP's. Due to their biocompatibility and low environmental impact, plant extracts have become a popular choice for making silver nanoparticles (Ag-NP's) [36].

**Figure 1:**
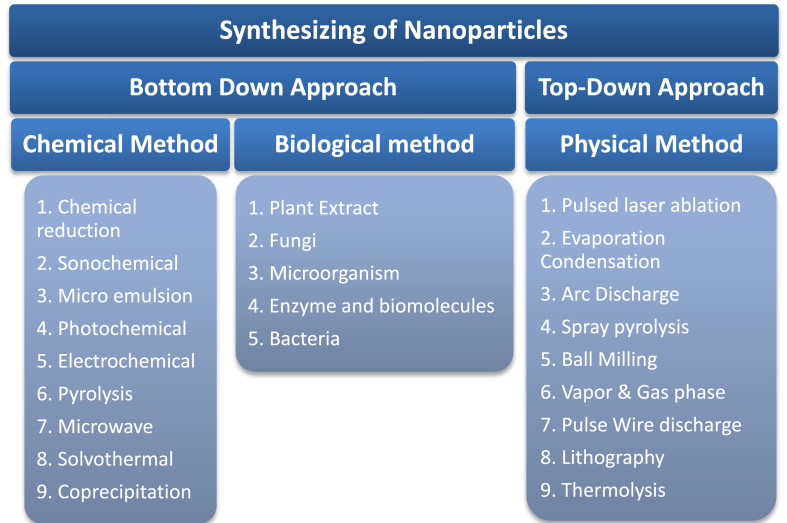
Developing nanoparticles through various methods [39].

In the process of biological production of silver nanoparticles, the organism functions as a capping agent, reducing agent, or stabilizing agent by converting Ag + to Ag0 [37]. As a result of their low price, high yields, and low toxicity to both humans and the environment, natural compounds generated from plants have become increasingly popular for use in biological processes in recent years [38]. Figure 1 shows the development of nanoparticles through various methods.

## 4 Chemical method

**Figure 2:**
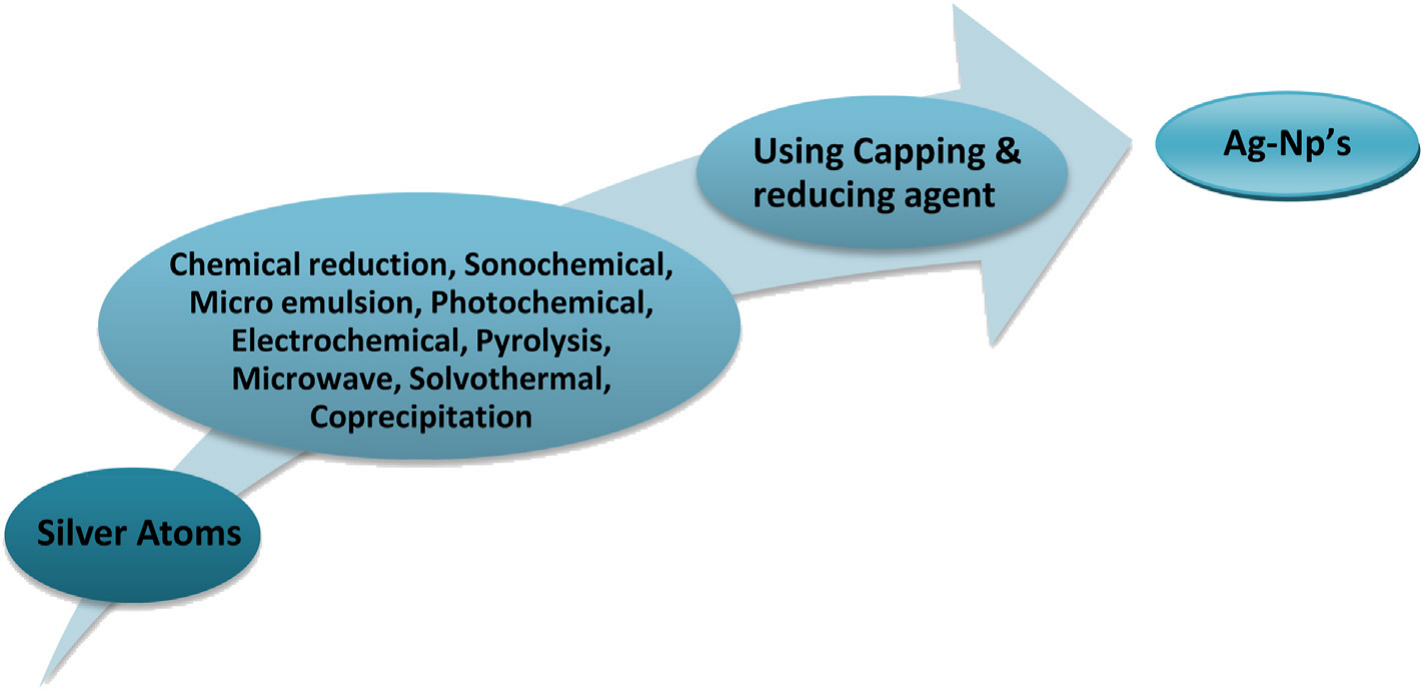
Chemical method of Bottom-down Approach for Ag-NP's Synthesis.

The tools needed for chemical approaches are typically less complicated and more easily accessible, which is a major plus. It is well known that the reducing agent plays a key role in converting silver ions to the metallic state, where they can then combine to create silver nanoparticles [40]. Size control and capping were accomplished with polyvinylpyrrolidone (PVP), and silver nanoparticles were stabilized with sodium borohydride and trisodium citrate [41]. Borohydride, 2-mercaptoethanol, citrate, and thio-glycerol are only a few of the chemicals and molecules employed in the manufacture of silver nanoparticles, and they are all extremely costly and potentially dangerous.Making silver nanoparticles of a specific size is quite challenging, and stopping them from sticking together needs extra work [42]. There is a great deal of potentially harmful and poisonous byproducts created during synthesis [43]. The main advantage of this technology is the synthesizing of nanoparticles with predefined form and size; nevertheless, environmental concerns have been raised due to the use of harmful chemicals, and severe reaction conditions such as high temp, pressure, and poisonous by-product [44, 45, 46]. Figure 2 shows the chemical method of bottom-down approach for the synthesis of AgNPs.

## 5 Physical method

**Figure 3:**
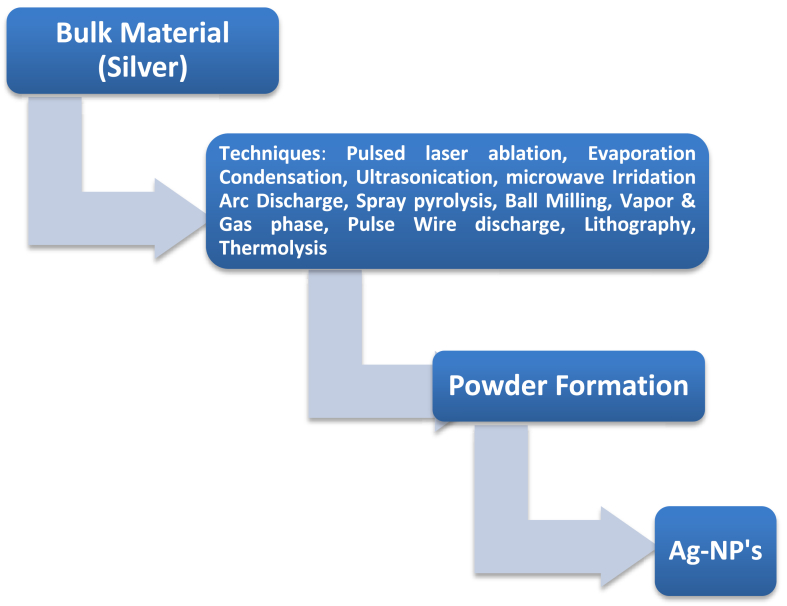
Physical method of Top-down Approach for Ag-NP's Synthesis.

Ultrasonication, microwave (MW) irradiation, and electrochemical procedures are examples of the "top-down" physical process used most often in the creation of nanoparticles by synthesis. The most common physical techniques include evaporation and condensation, as well as laser removal.The material is evaporated in a pontoon and then pointed at the burner to create a carrier gas [47]. It was discovered that polydisperse nanoparticles were produced when the heater surface temperature was held constant throughout time.These nanoparticles of silver were smooth and did not aggregate [48]. Plasma catalysis [49] and laser ablation [50, 51] are two examples of physical techniques.Conventional approaches are time-consuming and resource-intensive since they need the use of specialized equipment and the expertise of trained personnel.These techniques, as their names imply, involve the application of intense heat and light, both of which can be harmful, and the created nanoparticles are less stable [52]. Physical methods have the advantages of being quick, requiring no harmful chemicals, and being able to employ radiation as a reduction agent.However, physical processes include drawbacks such as wasteful energy use, uneven product distribution, and the presence of solvents in the final product [53]. Figure 3 shows the physical method of top-down approach for the synthesis of AgNPs.

## 6 Biosynthesis method

**Figure 4:**
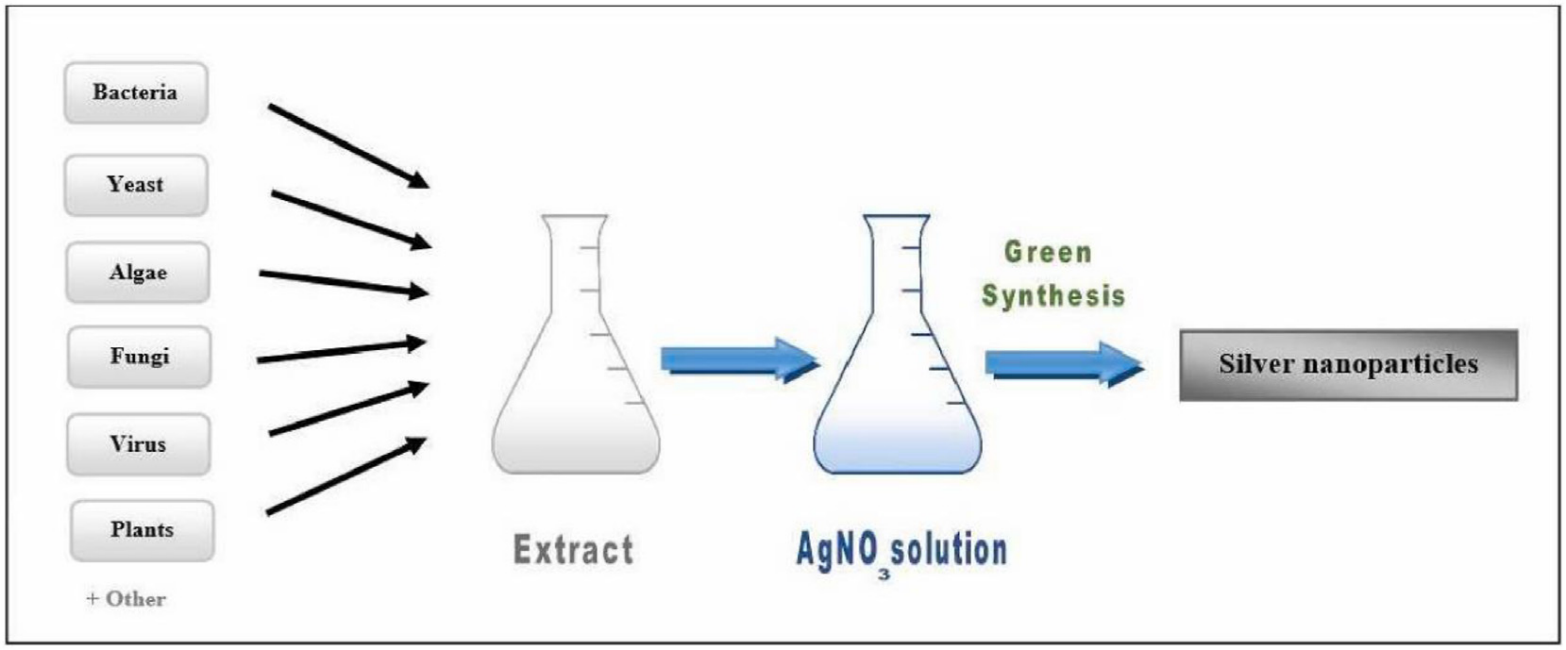
AgNP's extraction by biosynthesis method [54].

Implementing physical and chemical procedures to create silver nanoparticles is not only inefficient but also harmful to the environment. Therefore, it is crucial to create a system that is both cost-effective and safe for the environment, one that does not make use of dangerous chemicals [51] and voids the hazards of chemical and physical manufacturing methods. These gaps can be filled by biological approaches, which have several uses in health management due to their ability to regulate a wide range of biological processes. The employment of microorganisms like fungi, bacteria, and yeast, as well as natural materials like plants, are all examples of biological manufacturing processes. All of these factors have contributed to the widespread adoption of this strategy for the medicinal use of nanoparticles [36]. Purification of Ag-NP's necessitates cell lysis, adherence of microorganisms on nanoparticles' surface increases the risk of infection, and the microbial mode necessitates the search for potent strains producing Ag-NP's, growth and maintenance of microbial strain on expensive media, and less flexibility of pH and temperature during nano synthesis [1]. Plant material as a reducing agent in silver nanoparticle manufacturing has various benefits. In addition to being readily available, safe for handling, inexpensive, requiring almost little maintenance, and environmentally friendly are just a few of its many benefits [55]. Figure 4 shows the biosynthesis method for the extraction of AgNPs.

### 6.1 Mechanism of silver nanoparticles biosynthesis

**Figure 5:**
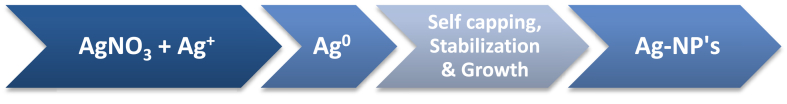
Ag-NP's (silver Nanoparticle) synthesis and related biochemical procedures [53].

Enzymes are the primary component of Ag-NP's production, whether it takes place within or outside of cells. Researchers have shown that nitrate reductase, which is dependent on the cofactor NADH, is essential for the production of AgNP. This enzyme helps nanoparticles form by moving electrons from the nitrate atom to the metal ion. It does this by acting as an electron shuttle [56, 57, 58, 59, 60]. The "factories" used to create silver nanoparticles are made out of a broad variety of plants, including therapeutic herbs. The potential mechanism of enzymatic AgNP production is typically comparable to that of microorganisms. However, compared to microorganisms, plant cells include a complex of varied antioxidant metabolites preventing the oxidation and degradation of cellular components. Therefore, the compounds used to stabilize and cap the nanoparticles are one-of-a-kind [61]. Due to their anti-inflammatory, antioxidant, anticancer, and other actions, they are especially crucial for the future of Ag-NP's in practical applications [62]. Evidently, "capping" agents can selectively attach to certain Nano crystal facets in order to alter the ratio of their respective surface areas by altering the corresponding surface free energy [63]. Thus, nanoparticle "capping" can perform various key activities, including preventing nanoparticle agglomeration, reducing toxicity, and improving antibacterial capabilities; also, these compounds can increase the bacterial cell affiliation and activity of Ag-NP's [64, 65]. Figure 5 shows the synthesis of AgNPs and related biochemical procedures.

### 6.2 Plant extract based Ag-NP's synthesis process

**Figure 6:**
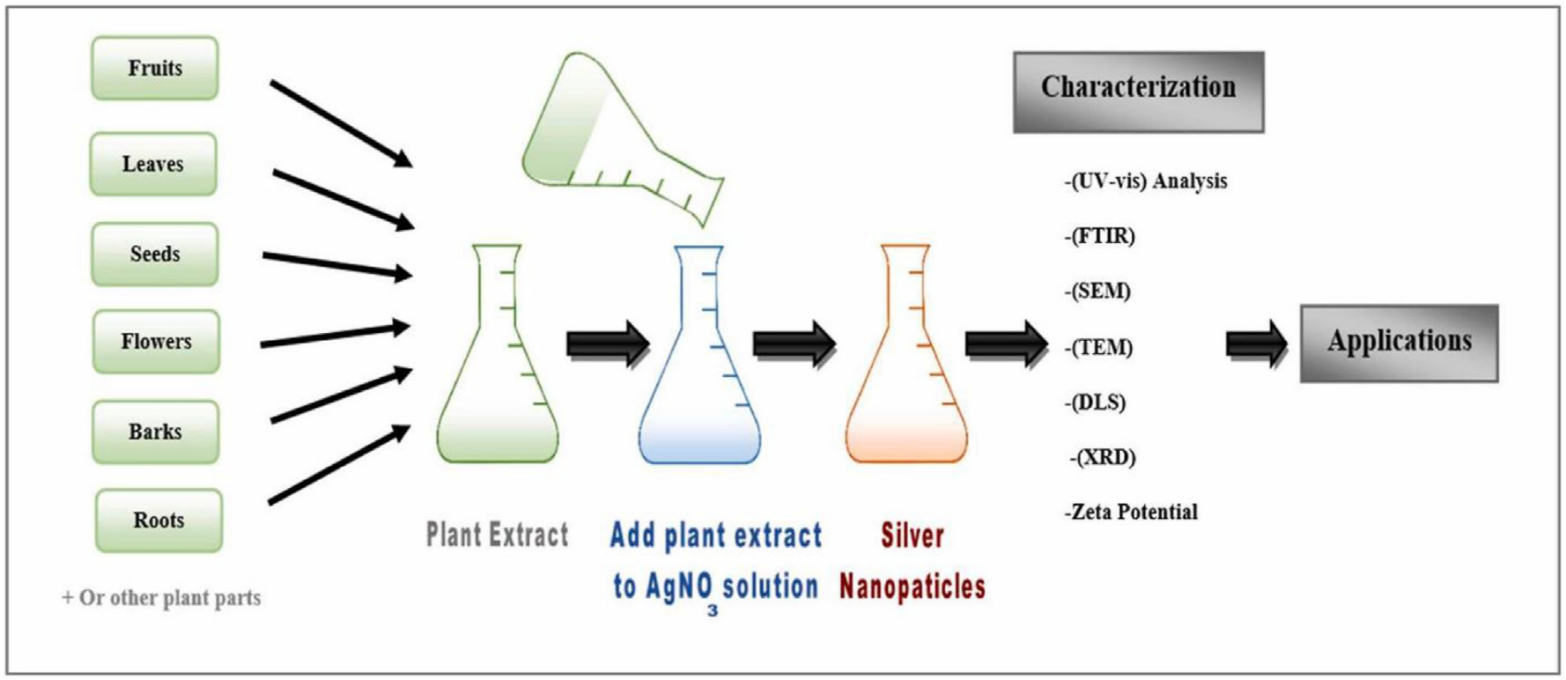
AgNP's extraction from plants by biosynthesis method [54].

Producing silver nanoparticles (Ag-NPs) in a live plant system was originally described in a study with *Alfalfa sprouts*, which was the first plant used in this way to produce metallic NPs [66]. Intracellular or extracellular plant-based synthesis can usually happen. The plant synthesizes intracellularly, whereas extracellularly in vitro. By using Phyto-compounds for precursor reduction, Ag-NP's may be synthesized directly from plant extracts through an extracellular route.Due to their biological potential, Ag-NP's are synthesized extracellularly from plant extracts. Scientific studies have shown that eliminating the extraction and purification steps makes plant extracts preferable for extracellular synthesis over intracellular synthesis [67, 68].Typically, a solvent solution, stabilizing agents, and reducing agents are needed for the extracellular production of Ag-NP's [69]. Phyto compounds in plant extract reduce and stabilize Silvernanoparticles.Since water is often utilized as the solvent solution, synthesis of Ag-NP's is thought of as environmentally friendly [31, 70]. The complex process of maintaining cell cultures is simplified by using plants and plant extracts in nanoparticle manufacturing, and this is seen as a benefit over using microorganisms [67]. In the presence of plant extracts, Ag-NP's take on two distinct morphologies throughout the process: the highly reactive face-centered cubic (fcc) structure, and the more strongly stabilized spherical form. The activity of Ag-NP's in a wide range of biological settings is well established, and it is known that their preferred development in the (111) plane and spherical form is responsible for this [69, 71, 72, 73, 74, 75, 76, 77, 78]. Figure 6 shows the biosynthesis method for the extraction of AgNPs from plants. Table 1 shows the synthesis of AgNPs from different plants.

**Table 1:** A list of Plant-Based Synthesized Ag-NP's ranging in size from 1-100nm and having spherical and FCC (face-centered cubic) shapes [38, 79, 80].

Scientific Name	Common Name	Plants Extract	Precursor	Shape	Size (nm)	Characterization technique	Ref.
*Angelicaepubescentis*	Female ginseng	Root	AgNO_3_	Almost spherical	12.48	UV-Vis Spectroscopy, SPR analysis, FE-TEM	[81]
*Coriandrum sativum*	Coriander	Leaf	AgNO_3_	Spherical	37	UV-Vis Spectroscopy, FESEM/EDX, FTIR, XRD, BET analysis,	[82]
*Benzoin gum*	Loban	Plant	AgNO_3_	Spherical	12–38	UV-Vis Spectroscopy, FE-TEM, FTIR, XRD	[83]
*Cucurbita maxima*	Squash	Petals	AgNO_3_	Roughly Spherical	76.1	UV-Vis spectroscopy, DLS, Zetasizer, FTIR, Fe-SEM	[84]
*Acorus calamus*	Calamus	Rhizome	AgNO_3_	Roughly Spherical	59.02 ± 1.3	UV-Vis spectroscopy, DLS, Zetasizer, FTIR, Fe-SEM	[84]
*Plumeria alba*	West Indian-jasmine	Flower	AgNO_3_	Spherical shape	36.19	UV-visible spectroscopy, DLS, SEM/EDX, FTIR, TGA/DSC, and XRD	[85]
*Abutilon indicum*	Indian lantern-flower	Leaf	AgNO_3_	Spherical	5–25	UV-Spectroscopy, Zeta Sizer, DLS, TEM, EDAX, SEM, XRD, FTIR, DSC	[86]
*Solanum trilobatum*	Purple fruited pea	Fruit	AgNO_3_	Spherical	12.50–41.90	UV-Spectroscopy, TEM, EDAX, SEM, XRD, FTIR	[87]
*Rosa indica*	cyme rose	Petals	AgNO_3_	Spherical	23.52–60.83	UV-Spectroscopy, TEM, EDX, SEM, XRD, FTIR	[88]
*Rosa damascena*	damask rose	Petals	AgNO_3_	Spherical	74–94	UV-Spectroscopy, DLS, XRD, FTIR, HRTEM, FE-SEM, EDAX	[89]
*Achillea biebersteinii*	Yellow milfoil	Flowers	AgNO_3_	Spherical	12 ± 2	UV-Spectroscopy, DLS, TEM, EDX	[90]
*Piper longum*	Indian long pepper	Fruit	AgNO_3_	Spherical	46	UV-Spectroscopy, DLS, FTIR, SEM.	[91]
*Artemisia tournefortiana*		Aerial part	AgNO_3_	Spherical	22.89 ± 14.82	UV-Spectroscopy, DLS, XRD, FTIR, HRTEM, EDX	[92]
*Artemisia marschalliana*	Field wormwood	Aerial part	AgNO_3_	Spherical	5–50	UV- Vis spectroscopy, SEM, TEM, XRD, FTIR	[93]
*Amomum villosum*	Malabar cardamom	Fruit	AgNO_3_	Spherical	5–15	UV-Spectroscopy, DLS, XRD, FTIR, HRTEM, EDX SAED	[94]
*Excoecariaagallocha*	River poison tree	Leaf	AgNO_3_	Spherical	23 and 42	UV-Spectroscopy, DLS, XRD, FTIR, FESEM, EDAX	[95]
*Cornus officinalis*	Asiatic dogwood	Fruit	AgNO_3_	Almost spherical	11.7	UV-Spectroscopy, DLS, XRD, FTIR, FESEM, EDAX	[96]
*Taraxacumofficinale*	Dandelion	Leaf	AgNO_3_	Spherical	5–30	UV-Spectroscopy, DLS, XRD, FTIR, HR-TEM	[97]
*Indigofera tinctoria*	Indian indigo	Leaf	AgNO_3_	Spherical	16.46	UV-vis. spectroscopy, FTIR spectroscopy, XRD, TEM, EDX, and AFM	[73]
*Syzygiumjambos*	Rose-apple	Leaf and bark	AgNO_3_	Spherical	5–8	UV-vis. Spectroscopy, TEM, XRD, FTIR, NMR	[98]
*Hydrastis canadensis*	Goldenseal	Whole plant	AgNO_3_	Spherical	90.87	UV- Vis spectroscopy, DLS, TEM, FTIR, AFM, XRD, CD	[99]
*Thuja occidentalis*	Arborvitae	Plant	AgNO_3_	Spherical	90.87	UV- Vis spectroscopy, DLS,TEM, FTIR, AFM, XRD, CD	[99]
*Mentha arvensis*	wild mint	Leaf	AgNO_3_	Spherical	3–9	UV- Vis spectroscopy, EDX, DLS, FTIR, AFM, XRD	[100]
*Anthemisatropatana*		Plant	AgNO_3_	Spherical	38.89	UV-Vis Spectroscopy, TEM, SEM, XRD, FTIR	[101]
*Ficus religiosa*	Peepul tree	Leaf	AgNO_3_	Spherical	21	UV-Vis Spectroscopy, TEM, FTIR, GCMS, DLS	[102]
*Sesbania grandiflora*	Vegetable hummingbird	Leaf	AgNO_3_	Spherical	10–25	UV- Vis spectroscopy, EDX, TEM, FTIR, AFM, XRD	[103]
*Syzygiumcumini*	Java plum	Leaf	AgNO_3_	Spherical	5–30	TEM,SAED, XRD, EDX	[104]
*Phytolacca decandra*	Pokeweed	Dried plant	AgNO_3_	Spherical	90.87	UV- Vis spectroscopy, DLS, TEM, FTIR, AFM, XRD, CD	[99]
*Gelsemium sempervirens*	Evening trumpet flower	Dried plant	AgNO_3_	Spherical	90.87	UV- Vis spectroscopy, DLS, TEM, FTIR, AFM, XRD, CD	[99]
*Allium sativum*	Garlic	Fruit	AgNO_3_	Spherical	3–6	UV-Vis Spectroscopy, TEM,XRD, EDX, FTIR	[105]
*Zingiber officinale*	Ginger	Fruit	AgNO_3_	Spherical	3–22	UV-Vis Spectroscopy, TEM,XRD, EDX, FTIR	[106]
*Origanum vulgare*	Oregano	Leaf	AgNO_3_	Spherical	63–85	UV- Vis spectroscopy, FE-SEM, DLS,XRD, FTIR	[107]
*Eucalyptus globulus*	Tasmanian bluegum	Leaves	AgNO_3_	Spherical	1.9–4.3 and 5–25	UV-visible spectroscopy, XRD, TEM, SEM-EDX, FTIR	[108]
*Phyllanthus niruri*	Gale of the wind	Leaves	AgNO_3_	Crystalline FCC and spherical	30–60	UV-visible spectroscopy, XRD, SEM-EDX, FTIR	[109]

*Bryophyllum and Asiatic Pennywort*		Leaves		Crystalline FCC and spherical	18–21	UV-visible spectroscopy, XRD, TEM	[110]
			AgNO_3_				
*Eucalyptus leucoxylon*	White ironbark	Leaves	AgNO_3_	FCC and spherical	50	UV-visible spectroscopy, XRD, SEM, TEM,	[111]
*Desmodiumtriflorum*	Threeflowerticktrefoil	Plant Broth	AgNO_3_	Spherical	10	UV-visible spectroscopy, XRD, TEM	[112]
*Achyranthes aspera L.*	Devil's horsewhip	Leaves	AgNO_3_	Spherical	1.0 ± 18.3	UV-visible spectroscopy, TEM	[113]
*Calotropis procera*	Giant-milkweed	Latex serum	AgNO_3_	Spherical	12.33	UV-visible spectroscopy, XRD, TEM, FITR	[114]
*Allium cepa*	Onion	Plant	AgNO_3_	Spherical	33.6	UV-visible spectroscopy, DLS, TEM	[115]
*Prunus persica*	Peach gum	Peach gum powder	AgNO_3_	FCC	23.56 ± 7.87	FITR	[116]
*Solanum lycopersicum*	Tomato	Fruit	AgNO_3_	Spherical	10	UV-visible spectroscopy, FITR, SEM, TEM	[117]
*Juglans Regia L.*	Walnut	Leaves	AgNO_3_	Almost spherical	10–50	UV-visible spectroscopy, TEM	[118]
*Chenopodium album*	Lambsquarters	Leaves	AgNO_3_	Almost spherical	10–30	UV-VIS, XRD, EDX, FITR, TEM	[119]
*Mentha piperita*	Pudina	Leaves	AgNO_3_	Spherical	5–30	SEM, FITR	[120]
*Catharanthus roseus*	Madagascar periwinkle	Leaves		Crystalline FCC and spherical	20	UV-vis spectrum, XRD, FTIR	[121]
			AgNO_3_				
*Terminalia arjuna*	Arjun	Bark	AgNO_3_	Spherical	2–100	UV-vis spectroscopy, FT-IR, XRD, SEM, and DLS	[122]
*Vitex negundo*	Chinese chaste tree	Leaves	AgNO_3_	FCC and spherical	5 and 10–30	UV-vis spectroscopy, TEM, XRD	[123]

### 6.3 Why it is better than other processes

In comparison to the employment of chemical, physical, and microbiological approaches, plant extracts for the production of Ag-NP's offer various benefits. Extracts from plants can be used to create nanoparticles without the need for hazardous reducing and capping chemicals, radiation, high temperatures, a specific microbial strain, or an expensive growing medium. Antimicrobial, medication delivery, and water purification are all examples of applications where microbial synthesis presents risks of infection and contamination throughout the synthesis process [124, 125, 126]. It takes a lot of time and effort to keep a pure culture of bacteria going so that they can synthesize a compound. Nanoparticle creation in plants is more rapid and they are immune to these limitations [124, 125, 126]. According to Botanic Gardens Conservation International (BGCI), there are around 321,212 plant species on earth. As a result, scientists have had access to a sizable class of plants to synthesize nanoparticles. Phytochemicals and other plant derivatives are among the wide category of plant extracts with the dual capabilities of reducing and stabilizing agents. Since the phytochemicals' –OH groups and carbonyl groups have been oxidized, they may serve as a reducing agent and a capping agent for the stability of nanostructured materials [27]. Because the availability of the reducing agent is greater in the extract than in the whole plant, aqueous plant extracts are typically used in biogenic synthesis for the manufacture of noble nanoparticles [127]. Waste materials and the synthesized goods themselves both originate from plant extracts found in nature, making this method more eco-friendly overall [128]. This bio-based technique for synthesizing nanoparticles allows for improved repeatability of the process and enhanced stability of the generated nanoparticles. Thus, this plant-based nanoparticle synthesis is suited for industrial-scale production with more efficient cost investment; it is also environmentally benign and safe for therapeutic application in humans [129].

## 7 Characterization techniques

In order to comprehend and regulate the NPs production process, it is crucial to conduct a thorough characterization of the Ag-NP's [130]. For instance, the agglomeration, spatial reasoning, geometric proportions, Brownian motion, intercalation, and scattering of NPs can only be determined by characterizing Ag-NP's. Elements, particle sizes, crystal size, porosity, solubility, surface characteristics, water sorption, and surface morphology are some other parameters that need to be analyzed [131, 132, 133].

## 8 UV-Vi's spectrophotometry

The creation of nanoparticles may be confirmed and their stability monitored by UV visible spectrometry measurement, which is the most frequent quantitative method. The technique of UV visible spectroscopy is quick, sensitive, and easy to use. Additionally, it may choose and choose amongst several nanoparticles. After completion of synthesis, a brownish yellow color was seen in the AgNO3, and absorption spectra appeared within 410 and 430 nm. In other words, it provides a numerical representation of the sample's solution's exposure to ultraviolet (UV) and visible light [79].

## 9 X-ray diffraction analysis (XRD)

Based on the insight it provides into the degree of crystallinity, it is frequently employed in the research of nanoparticles, biomolecules, polymers, and other similar substances. It provides a rough estimate of the various chemical groups and particle sizes and provides quantitative information on the resolution of various chemical compounds. This research is based on the fact that various crystals will generate unique diffraction patterns when subjected to a monochromatic X-ray beam. Using Bragg's equation, the interference pattern created by these diffracted X-rays may reveal whether a material is crystalline or polycrystalline. Measurements from this method are expressed in Angstroms (Å) (1 Å = 0.1 nm) [79].

**Figure 7:**
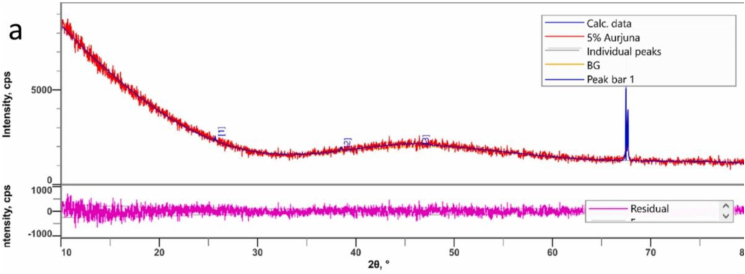
Example of XRD image of synthesized bioplastic at (a) 5% Arjuna [134].

The transmission electron microscope (TEM) is the most widely used and effective method for studying silver nanoparticles. It is often employed to get the size and shape-related quantitative parameters of Ag-NP's.The sample is subjected to an electron beam, and the resulting interaction is captured on a photographic plate. Due to its tremendous resolving power, TEM can identify and measure even the tiniest of nanoparticles.Compared to SEM, TEM has superior spatialre solution power. Multiple high-resolution microscopy techniques that use a stream of high-energy electrons are now being developed by scientists in this field of nano-science and technology [79]. Figure 7 shows an XRD image.

## 10 Scanning electron microscopy (SEM)

**Figure 8:**
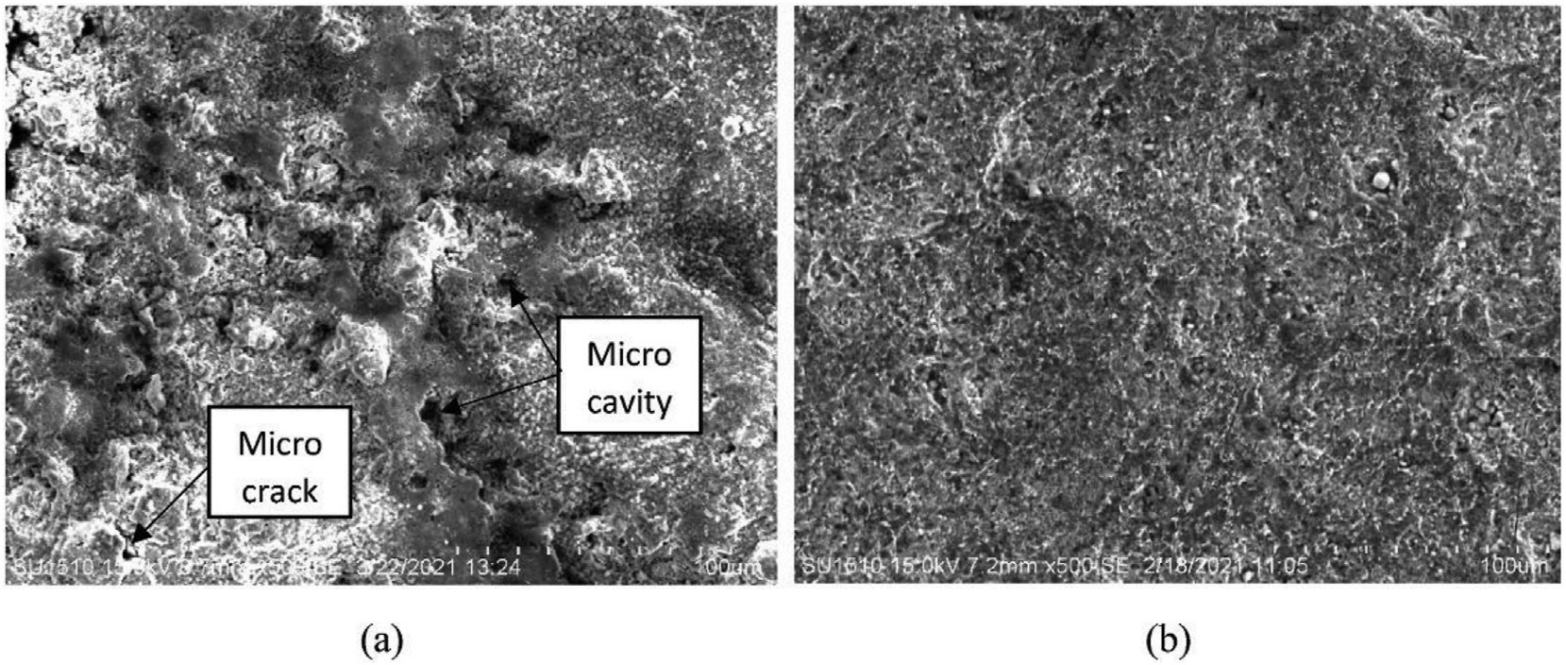
Example of SEM image of mild steel surface (a) uninhibited solution, (b) inhibited solution [135].

Scanning electron microscopy (SEM) uses the repulsion and attraction of electrons between beams directed through a sample and atoms at various depths to create images. Detectors record the secondary electrons that have been reflected and convert them into visuals. Right now, scanning electron microscopy (SEM) is the best microscopy approach for identifying and characterizing nanoparticles of varying sizes and shapes. Data concerning the nanoscale surface morphology of particles can also be determined. SEM's use is favored because of its ability to resolve particles smaller than 10 nm [79]. Example of SEM image can be seen in Figures 8(a) and 8(b).

## 11 Dynamic light scattering (DLS)

Nanoparticles in suspension can have their size distribution and poly-disperse nature analyzed by dynamic light scattering (DLS).In most cases, this is the method of choice when attempting to ascertain a size. This approach estimates the hydrodynamic radius of a particle in Brownian motion, which is the key to understanding how it works. When a particle in the solution is hit with a laser beam, it scatters the light at varying intensities. Particle sizes were calculated by analyzing the fluctuations' intensities using the Stokes-Einstein relationship equation. From 20-200 nm, DLS consistently measures particle sizes [79].

## 12 Fourier transform infrared spectroscopy (FTIR)

Chemical bonds and functioning molecules on a surface can be evaluated by FTIR spectroscopy. Additionally, it may be used to analyze the physical characteristics of artificially created nanoparticles to identify the biomolecules involved in nanoparticle creation. It was utilized by researchers to identify the chemical ingredient employed in the creation of nanoparticles in leaf or other plant extracts. The principle behind this method is that whenever infrared radiation strikes a sample, a portion of its energy is consumed and the rest is released. By analyzing the sample's absorbance and transmittance values, the spectrum may identify the sample. To investigate the effect of plant extract in the reduction of silver, FTIR is a non-intrusive, appropriate, significant, and incredibly easy technology [79].

The FTIR spectra shift due to the presence of carbohydrates and phenolic compounds in the pure plant extracts. Carbohydrates, phenolic compounds and flavonoid compounds present in the pure plant extracts reduce the Ag ion and form AgNPs. Besides, they are also responsible for the stabilization of AgNPs and increases antimicrobial activities [136].

## 13 Energy-dispersive X-ray spectroscopy (EDX)

Applications of EDX in nanotechnology have been well documented as an essential tool for determining a sample's elemental makeup.The X-ray spectra of any given nanoparticle may be used to determine its elemental makeupsince each element has a distinct atomic structure that results in a distinct collection of peaks [137].

## 14 Transmission electron microscopy (TEM)

**Figure 9:**
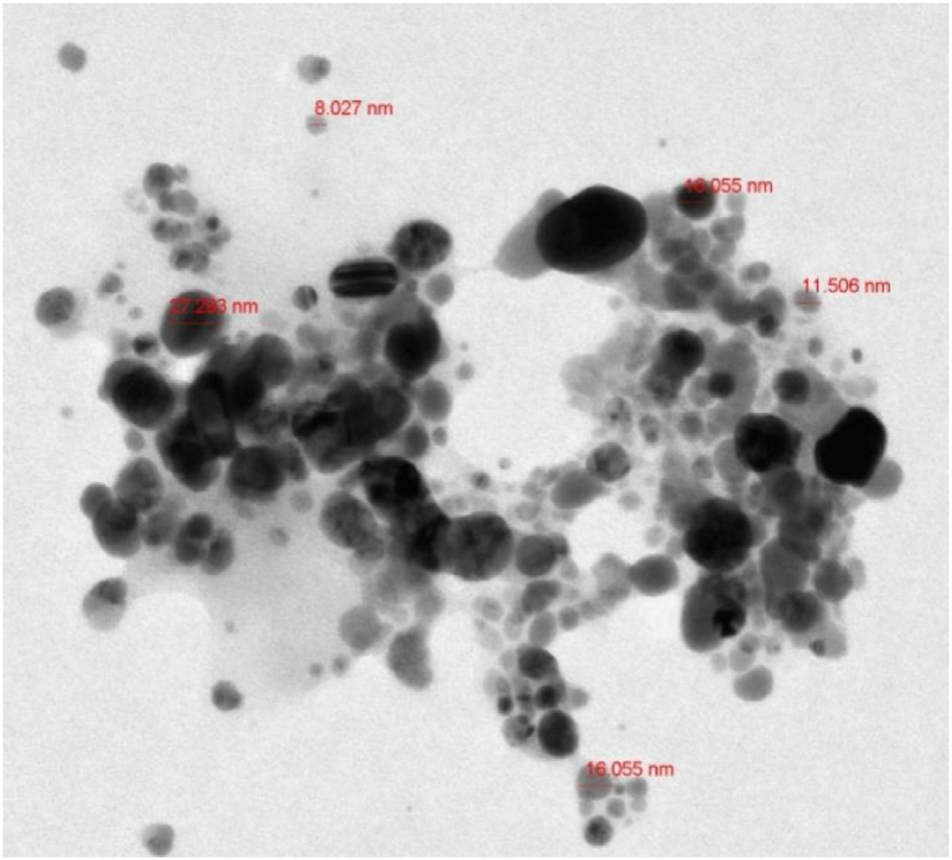
Example of a TEM image of AgNPs synthesized by *A. fleurentiniorum* extract [142].

Transmission electron microscopy involves passing a stream of electrons through a very thin specimen, where they interact with the object and create a picture [80]. As part of getting AgNPs ready for TEM investigation, a trace quantity is spread onto carbon-coated copper grids. The AgNPs are embedded in an ultrathin, dried grid that a beam of light travels through and reacts to [138]. This picture is formed as a result of an interaction between electrons passing through the AgNPs. A magnifying lens or other imaging equipment records a blown-up version of the scene [139]. The antibacterial capabilities of AgNPs can be improved by the use of medicinal plants in their synthesis, and the size and shape of the resulting AgNPs can be controlled in this process as well [140, 141]. An example of TEM image can be seen in Figure 9.

## 15 Auger electron spectroscopy (AES)

As an outcome of the collisions between an electron beam and the atoms permanently stationed at a sample's surface, atomic emission spectroscopy (AES) is a surface-sensitive analytical method [143]as well as a top-notch nanotech analysis tool [144].

## 16 Low-energy ion scattering (LEIS)

It is generally known that LEIS has the highest sensitivity to the surface of any technology now in use for surface analysis.The approach allows one to determine the structure and elemental content of a material [145, 146, 147]. Strong LEIS is an essential surface analysis technique that plays a significant role in the analysis of SAM-functionalized nanomaterials [148].

## 17 Applications of Ag-NP'sin dental implant

The antibacterial and antifungal uses of Ag-NPs are extensive. Numerous dental composites feature an antimicrobial coating made of silver nanoparticles (Ag-NPs) [149]. Multiple dental treatments can benefit from using silver nanoparticles (Ag-NP's), which have been extensively studied for their antibacterial properties. Acrylic resins, Nanocomposites, adhesives, resin comonomers, intracanal medicines, and implant coatings have all been shown to benefit from Ag-NP's' potent antibacterial action in in vitro experiments [20].

Adding Ag-NP's to dental composites has been shown to improve their antifungal properties, while another study indicated that doing so decreased microbial colonization of dental implant covering materials [150]. Because of their inability to promote infection, devices based on silver nanoparticles are frequently employed in dental implants. Researchers have demonstrated that silver nanoparticles are efficient against Gram-negative and Gram-positive bacteria [151, 152]. The antibacterial activity of Ag_2_O nanoparticles prepared from the root extract of *Ficus benghalensis* was evaluated in vitro against dental bacterial strains [153].

Researchers found that silver nanoparticles made using aqueous *Mangifera indica* leaf powder have the potential to be used as dental fillings due to their flake-like form and small size (32.4 nm).Glass ionomer cement (GIC) was used to increase the mechanical characteristics of the created silver Nano biomaterials, which in turn increased their resistance to bacteria like *S.aureus* and *E. coli*. Combining these Nanoscale bio material-reinforced GIC with traditional dental implant materials as a composite or coating them on the surface may increase their mechanical strength [154]. Silver nanoparticles produced with *Oleo europaea* extract (white pepper oleoresin) showed increased antibacterial activity against oral infections. Because of their ability to inhibit microbial growth, they can be useful as a surface modification for traditional dental implants [155, 156, 157].

Dental implant insertion is complicated by alveolar bone loss, a frequent medical issue. In order to successfully restore the alveolar ridge, it is imperative that a barrier be placed in between bone graft and thus the gingiva. This will prevent the regeneration of fibrotic tissue, reduce the risk of bacterial infection, and stimulate bone growth. A study showed that using AgNP-coated collagen membranes after implanting bone grafts in alveolar ridge repair can reduce the risk of infection [158].

A study shows that 30 nm-sizedAgNP's doped with Ti Alloy showed better antimicrobial and biocompatibility [159]. As a surface modifier of dental implants, Ag-NP'scoated Ti showed better antimicrobials against *S. mutans* and *P. gingivalis* [160].

**Figure 10:**
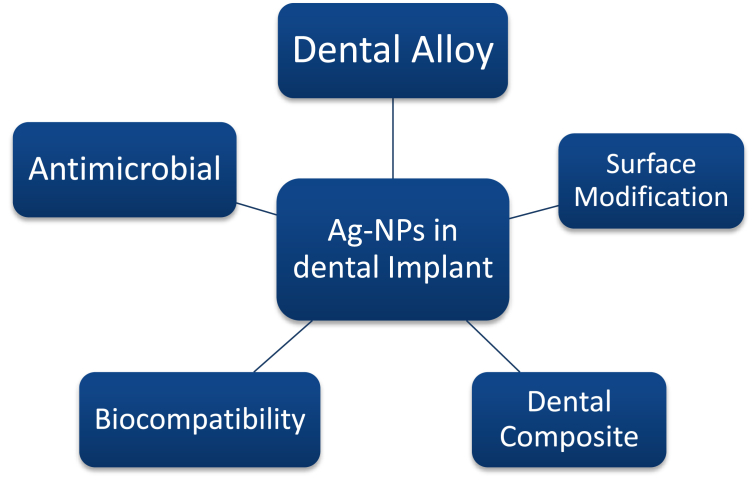
Ag–Np's Applications in dental Implants.

All of thisresearch showed that Nano biomaterials have great potential for use in dental implants.Potentially, innovative Phyto Nano biomaterials for dental implants might be more easily manufactured as a result of developments in contemporary synthesis procedures [155, 156, 157]. Applications of AgNPs in dental implants can be seen in Figure 10.

## 18 Current challenges and future prospects

Nano biomaterials made from plants are showing promise as a surface modification for dental implants, and related in vitro research is gaining traction.However, there are also several obstacles and major shortfalls in plant-mediated Nano biomaterial manufacturing.While bionanomaterials produced by plant-mediated synthesis tend to be more stable than those produced via more traditional methods, their speed of production makes them less ideal for some applications [157].

• Small Ag-NP's (d < 10 nm), according to the results of a quantitative investigation, display strong suppression of antibacterial activity due to its increased release of Ag-NP's ions. However, they centrifuged a solution with a very high concentration of bigger Ag-NP's, removed the supernatant containing the Ag-NP's ions, and then utilized just the pellet fraction containing the particles. The results are on par with those of smaller Ag-NP's. Based on these findings, we can only surmise that there are certain characteristics of nanoparticles that are responsible for their antibacterial action, and more work can be done in improving those certain characteristics [14].
• Aggregation of Ag-NP's is a major issue since it reduces their surface area to volume ratio and, therefore, their activity in several sectors (antimicrobial, catalyst, etc.). Because it contains powerful reducing and capping agents, the phytochemical approach can be used to solve this issue as well. The future potential of phytochemical synthesis of nanoparticles lies in the manipulation of plant genomes using genetic engineering technologies in order to increase the production of active principles responsible for the reduction and capping agents [1].
• Delamination or nanoparticle release from modified implants can trigger a cytotoxic reaction, but minimal efforts have been undertaken to assure the effective production of nano-engineered coatings on commercial implants with sufficient mechanical stability [15, 16, 17].
• Bio-fabrication of Ag-NP's via plant extracts is a hot topic in the scientific community right now, although the specific mechanism involved in this process is not extensively researched. The processes involved in the production of Ag-NP's from plant extracts have only been speculated upon by a few researchers so far [31, 70]. Because of the wide variety of phytoconstituents that are present as well as the variation of plant extracts, it can be challenging to identify the specific stabilizing and reducing agent that is responsible for the formation of nanoparticles as well as their ability to maintain their stability [161].
• Up until this point, it was believed that phytocompounds such as proteins, organic acids, and secondary metabolites such as terpenoids, flavonoids, and phenolic acid could be used effectively as stabilizing and reducing agents in the bio fabrication of silver nanoparticles (Ag-NP's). However, it is more likely that several Phyto compounds found in plant extracts work in concert to reduce metal precursors [161, 162].
• It should be highlighted that the Nano biomaterials created recently for dental applications using plant extracts are freestanding Nano biomaterials. This means that in the near future, nano biocomposites may be fabricated using plant extracts, which is great news for a broad variety of commercial uses, such as surface-modified dental implants with special biological properties [157].

Last but not least, there are numerous papers and reviews discussing ways to enhance plant-extracted Ag-NP's, including how to lessen their toxicity, increase their effectiveness, enhance their antibacterial activity, etc. However, there is less research on how these plant-extracted Ag-NP's are specifically enhanced in the application of dental implants, suggesting that this area needs more study.

## 19 Conclusion

Since the beginning of modern dentistry, dental implants have been recognized as a reliable method of providing efficient medical therapy. Researchers consistently engage in comprehensive research intending to advance the dental implant industry. In order to accomplish this goal, nanoparticles have brought about a highly practical advancement in this area. When compared to other nanoparticles, silver nanoparticles (AgNPs) stand out as an excellent choice since they possess a variety of advantageous qualities. It has been used in a variety of dental implant disciplines. Ag-NPs can be utilized for antibacterial activity, biocompatibility, improved strength, and surface modification. In the realm of nanoparticles, factors such as form, size, and toxicity hold a significant amount of importance. These factors are necessary for the action of Ag-NP. In recent years, researchers have been making efforts to acquire silver nanoparticles (Ag-NPs) from a variety of sources, with the goals of achieving the desired shape and size in order to enhance the material's activity in dental implants, as well as acquiring them in a less toxic manner and a less toxic type. Furthermore, the synthesis of plant extracts is one of the most viable approaches for obtaining these highly effective Ag-Nps. Despite the vast amount of published research on plant-extracted silver nanoparticles and their application in dental implants, there is still a need for research into the subject of optimizing the form, size, and purity of plant-extracted silver nanoparticles for use in dental implant applications. In the end, we can say that plant-extractable Ag-NPs have a bright future thanks to advancements in synthesis, and they also have enormous utility in the dental implantation industry.

## Declarations

### Author contribution statement

All authors listed have significantly contributed to the development and the writing of this article.

### Funding statement

This research did not receive any specific grant from funding agencies in the public, commercial, or not-for-profit sectors.

### Data availability statement

Data will be made available on request.

### Declaration of interests statement

The authors declare no conflict of interest.

### Additional information

No additional information is available for this paper.
